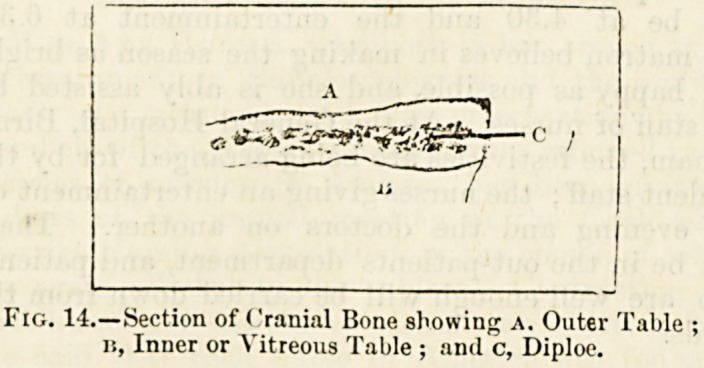# The Hospital. Nursing Section

**Published:** 1901-12-14

**Authors:** 


### The Hospital.

**Nursing Section.**

Contributions for this Section of "The Hospital" should be addressed to the Editor, "The Hospital" Nursing Section, 28 & 29 Southampton Street, Strand, London, W.C.

No. 794.—Vol. XXXI. *SATURDAY, DECEMBER 14, 1901.*

### Notes on News from the Nursing World.

### THE ROYAL RED CROSS.

The King has conferred the decoration of the Royal Red Cross upon Miss Agnes Mary Waterhouse and Miss Marian Jeannette Hislop, nursing sisters, in recognition of their services to the sick and wounded during the operations in China. Miss Hislop was trained at St. Bartholomew's Hospital, where she was subsequently staff nurse. From October 1888 to June 1889 she was sister at the Hospital for Sick Children, Great Ormond Street. In 1890 she joined the Indian Nursing Service, of which she is now senior sister. Soon after the operations in China commenced she joined the Expeditionary Force at Wei-hai-wei.

### THE PRINCE OF WALES AND HIS NURSE.

With characteristic thoughtfulness the Prince of Wales expressed his personal wish that "Sister Victoria," of St. Mary's Hospital, who, as our readers will remember, has on two occasions nursed him during his illness, should be invited as one of the guests of the Corporation at the Guildhall on Thursday. An act of real kindness like this might well be emulated. Hospital nurses do not have many opportunities of taking part in the events going on in the outside world. Of course, a reception to Royalty in the City of London is not an everyday occurrence, but there are other festivities to which a nurse might now and then be bidden. The compliment, and especially the spirit dictating it, would, we are sure, be greatly appreciated.

### OUR CLOTHING DISTRIBUTION.

As usual we give our kind friends who each succeeding year help us to extend our Christmas clothing distribution a final reminder. Monday next, December 16th, is the last day for sending in parcels. They should be addressed to the Editor of The Hospital, 28 and 29 Southampton Street, Strand, W.C., and the name and address of the sender should be enclosed in each parcel. In addition to the parcels already acknowledged we have received contributions from Miss M. McLennan, 20 Heriot Road, Edinburgh; Miss Rodgers, 82 Station Road, Epsom; Nurse Sykes, Warwickshire; Nurse Peter, Cross Deep, Twickenham; Nurse A. Hillman, Charmouth; and Miss F. Knight, 42 Anson Road, Tufnell Park, N.

### MEMORIAL TO ONE OF "THE PRINCESS OF WALES'S" NURSES.

On the morning of Advent Sunday a handsome window was finally dedicated in St. John's Church, Ravenhead, to the memory of Nursing Sister Clara Evans, who died at Bloemfontein. Miss Evans was one of the nurses of the London Hospital who were chosen by Queen Alexandra, then Princess of Wales, to proceed to South Africa for service during the war, and she fell a victim to the epidemic of enteric fever in the Orange River Colony last year. The window was specially designed by the three sisters of the deceased. It consists of three lights. One represents a South African battlefield, with Bloemfontein in the distance; another Miss Evans in army nursing costume; and the third is a picture of a ward in the "London" Hospital. Miss Evans was formerly a Sunday School teacher at Ravenhead.

### A WELL-EARNED DISTINCTION.

Miss Rosalie Harvey, who was lately awarded by the King the Kaiser-i-Hind silver medal for public services in India, of second class, is a missionary of the Zenana Bible and Medical Mission. The value of Miss Harvey's work during the recent visitations of plague and famine in the Nasik has thus been suitably recognised. Miss Harvey, it may be remembered, undertook the work of nursing in the plague hospitals during the winter of 1896–7, and in 1899, when there was another outbreak, she had charge of the native Christian Hospital at Sharanpur. She lately founded, and now maintains, a Home for Lepers at Nasik, which contains over 100 inmates.

### THE WAR NURSES.

Two detachments of nursing sisters disembarked at Southampton from South Africa on Thursday from the *Simla* and *Wakool* respectively. The following nurses composed the "ship's staff" on the *Simla*: A.N.S. Acting Superintendent M. E. Harding, who requires six weeks' leave before returning to South Africa; L. Langley Naylor, A.N.S.R.; A. M. Winder, A.N.S.R.; and M. Smythe, A.N.S.R., all of whom wish to return in ss. *Simla*; and L. P. Dixon, A.N.S.R., who requires one month's leave and returns to South Africa. On the *Wakool* were the following members of the Army Nursing Reserve, all of whom are returning to South Africa after one month's leave: A. Knaggs, M. G. Gilmore, V. J. Lamb, and F. Smallwood (whose length of leave is not specified). M. Franklin, Colonial Nursing Service, was also on board, and is returning to South Africa. It is announced that the Imperial Yeomanry Hospital at Bloemfontein will be transferred to the authorities on December 28th, and that the nursing staff will return to England by the first available transport.

### TRADUCING ARMY SISTERS.

The attacks which several anonymous correspondents have been allowed to make on "nursing sisters at the front" in the columns of the *Daily News* cannot be answered categorically because of their indefinite character. For example, in one of the letters it is stated that some time in the autumn of last year a sister told a patient, who appealed to her because he was suffering greatly from the dressings coming off after a serious amputation, that "the dressings had no business to come off, and that she had no time to attend to them." The same correspondent affirms that, in his own case, massage was ordered twice a day, "but the instructions of the medical officer were practically ignored," and he only received massage twice in nearly three weeks. He complained to the nurse and her reply, he says, was that "she had no time to massage his leg, though shortly after this occurred he saw her galloping across the veldt with other sisters and officers on horseback." But beyond the vague statement that he served as a volunteer "with a distinguished regiment," the man gives no indication of his identity. Either his story is true or it is false. If it be true, why does he not sign his name and make his charges definite? His final assertion, that "as a rule the services rendered by the sisters in the hospitals through which he passed were of comparatively little value to the sick and wounded men," is, however, so obviously a sweeping slander that we are not surprised he is afraid to disclose his personality, even in writing to a paper whose sympathies are pro-Boer.

### NURSING AT NAAUWPOORT.

Madame Alice Bron has already expressed in The Hospital her high appreciation of the manner in which Boer prisoners are treated in English hospitals in South Africa, and in an account which she has just given to a foreign contemporary of her experiences in the English nursing service she cites a striking instance of the consideration shown to the wounded enemy. "After capital operations the patient," she says, "has champagne of the choicest French brands in considerable quantity, then old cognac, and finally port, stout, or ale at choice, with five or six eggs a day beaten up in brandy and milk, arriving at last at a complete diet of which I, though perfectly well, could not have absorbed the half." This is all very well, but another picture which Madame Bron draws is a striking contrast:—"During the winter I passed at Naauwpoort, 5,000 feet above the sea, where the cold is awful, do you think that there were any fires in barracks or in our rooms? Do you think that on night duty, when rounds have to be made in snowstorms, with sheets of rain and wind dashing out your lantern and compelling you to stand with your feet in pools of water while you light it—while all the time sniping is going on close at hand—to go afterwards to sit by the bedside of a dying man—do you think, I say, that that is comfortable?" Madame Bron does not complain of the life which, in common with other nursing sisters brought up in comfortable English homes, she has to undergo, but there can be no question about the endurance of hardness.

### THE CASE OF MISS HOBHOUSE.

Even if Miss Emily Hobhouse had not initiated legal action in respect to her deportation from South Africa, we should not in these columns discuss the general question. But as some of our readers have asked whether the lady who states that she set out for the concentration camps with the view of nursing some of the sufferers is a trained nurse, we may say that she is not. That there should be a contrary impression is, however, not surprising, since the address of Miss Mabel Ella Hobhouse, who was trained at Guy's Hospital, and was afterwards sister, is a Wiltshire lady. Miss Emily Hobhouse has not, so far as we are aware, claimed to possess professional qualifications. As to the alleged refusal of Sisters MacKillan and Nicholson to forcibly remove Miss Hobhouse to the *Roslin Castle*, we understand that such a thing was not contemplated, and that these ladies were merely requested to conduct her to the transport. It was naturally inferred that Miss Hobhouse would sooner go quietly in the company of two of her own sex than under the escort of soldiers.

### THE ROMANCE OF THE WAR.

We understand that on New Year's Day Miss Grace Tillott, who nursed the sick and wounded in South Africa for more than twelve months as a member of the Army Nursing Service Reserve, will be married to Mr. Phillip Read, son of Sir Hugh Gilzean Read, who went to the front at the beginning of the campaign with the first Warwickshire contingent of the Imperial Yeomanry.

### DUNDEE AND SOUTH AFRICA.

In proportion to its size, Dundee Royal Infirmary has done well among provincial hospitals in sending nurses to South Africa. It has lately had the honour of having four nurses of its training selected for duty as matrons in the refugee camps. Sister Macgregor was home-sister; Sister Stevens had charge of the maternity and gynæcological wards before she took up private nursing a year ago; Nurse Mitchell was Queen's nurse for nine years; and Sister Mary F. McKenzie was for three years ward sister, and four years assistant matron. On leaving Dundee, Sister Mary received farewell gifts from the nursing staff and one from the domestic staff. Miss Mitchell and Miss McKenzie sailed in the transport *Canada* for South Africa.

### A TRIBUTE OF RESPECT.

Among the numerous floral tributes deposited round the grave of Sir William MacCormac on Monday was one from the matron, sisters, and nurses of St. Thomas's Hospital. Sir William was not only popular among the staff of St. Thomas's, but he was greatly esteemed by all nurses with whom he came in contact. He invariably had a pleasant word for them.

### DISTRICT NURSING IN AMERICA.

Miss Amy Hughes, who both in her speech at the entertainment to Buffalo delegates and the reply, in which she acknowledged the presentation by the members of the Nurses' Co-operation, referred to the systems of nursing in America, has been giving an address at the annual meeting of the Cheltenham District Nursing Association, in the course of which she again alluded to the subject. Contrasting district nursing in the States with district nursing here, she said that each nurse in America was too much inclined to branch off and deal with the people as she chose, while the idea of gathering the nurses into a central home and placing them under a head trained superintendent did not fit in with their notions of independence. Miss Hughes does not conceal her opinion that the method of allotting a district to a nurse and allowing her to live separately does not answer. As she pointed out, "in one case a nurse would overwork herself through too much zeal, while in another she would be too slack." We agree with her that it is a better arrangement "to have a home to which the nurses can come after their work for rest and encouragement from the superintendent, and where they can give others the benefit of their experience." Unfortunately, the encouragement is not always forthcoming, but the Home of the Cheltenham Association is admirably conducted, and has the cordial support of all classes in the town.

### THE NURSING QUESTION AT RANGOON.

Complaints are made of the inadequacy of the nursing staff at Rangoon Hospital. At present there are ten nurses and eight probationers attached to the institution, but it is urged that 32 are required, and a scheme for the employment of that number has actually been prepared by Major Davis, who raised the nursing question at a recent municipal meeting. The staff has been considerably augmented since it was taken over by the municipality, but if, as it is said, the increase has been even as much as 1,000 per cent. in the last 20 years, this is no excuse for the nurses being overworked now. There is no finality in the hospital world, and if patients continue to increase in the Rangoon Hospital, and the needs to multiply, the number of nurses must be also augmented, unless the municipal committee desire to be accused, with good reason, of neglect of their duty.

### CHRISTMAS AT THE HOSPITALS.

The Christmas entertainments at Charing Cross Hospital will be of the same character as they were last year; the tables in the wards will be decorated with flowers and plants, and there will probably be carol singing. But no definite arrangements were made, the matron said, when our representative called on Tuesday afternoon, except with regard to the puddings, which were all ready for the Christmas dinners. There will be great doings at the Chest Hospital, Victoria Park, and we understand that the house physician has been asked to prepare for the character of "Early Postman" or of "Father Christmas." There will also be fish-ponds or a bran-pie, and the nurses are preparing an exhibition of wax-works. The festivities will take place in one of the large corridors, and the bran-pie or fish-pond will be in one of the wards. Tea will be at 4.30 and the entertainment at 6.30. The matron believes in making the season as bright and happy as possible, and she is ably assisted by her staff of nurses. At the General Hospital, Birmingham, the festivities are being arranged for by the resident staff; the nurses giving an entertainment on one evening and the doctors on another. These will be in the out-patients' department, and patients who are well enough will be carried down from the wards.

### FISHERTON NURSING ASSOCIATION.

On Wednesday evening, last week, there was a considerable attendance at the Council Chamber, Salisbury, the occasion being a soirée for the benefit of the Fisherton Nursing Association. It was the first venture of the kind by the executive committee. There was an excellent musical programme, a supply of dainty refreshments, and an irreducible minimum of speech-making just to put strangers *au courant* with the good work of the institution. The report of the association shows that 1,864 visits were paid in a year to 150 houses; 49 serious cases were nursed.

### ST. LAWRENCE'S NURSES.

The Countess Cadogan has shown the interest she takes in the nursing movement in Ireland by attending the annual meeting of the supporters of St. Lawrence's Catholic Home in Dublin, an admirable society which provides trained nurses for the sick poor in their own homes. Some interesting details of the work of the organisation was given by the hon. secretary, who said that the tenth year of its existence had been signalised by a record which far surpassed anything that had been before achieved by the staff. Upwards of 900 cases were nursed, and considerably more than 14,000 cases visited. During the year the committee had trained four probationers, but these, it should be explained, receive a full hospital training before joining the Home. The course of training at the Home is for six months, and is necessary as a qualification for the position of a district nurse under the control of the Queen Victoria Jubilee Institute. It speaks well for the institution that there are 29 districts in Ireland where St. Lawrence's nurses are now supplying a great need in ministering to the wants of the poor and scattered population of the country.

### THE ABERYSTWYTH NURSING ASSOCIATION.

No nursing organisation can possibly make satisfactory progress unless it is managed in a businesslike manner, and we regret to see it stated that this is far from being the case in respect to the Aberystwyth and Llanbadarn District Association. It appears that at the annual meeting complaints were made that important business is transacted without consulting the committee, a state of affairs which, if not speedily altered, must soon affect the useful practical work of the Association. A more serious assertion was that the officials of the Association "treat the nurses as if they were scullerymaids." It may be observed that even scullerymaids are entitled to be treated with courtesy, but of course the trained nurses who are employed by the Aberystwyth Association have a right to look for the consideration which is usually given to ladies in their position.

### PENSION FUND SOUVENIR.

Owing to the number of orders received for the frames for the Souvenir plate, we regret that we must now withdraw our offer of last week. Those nurses who have already paid for frames will receive them in due course; but the cost of production is so heavy and the risks of breakage in transit so great that we cannot continue the supply.

### SHORT ITEMS.

The course of winter entertainments for in-patients at the National Hospital for the Paralysed and Epileptic, Queen Square, Bloomsbury, commenced on Thursday evening, when an excellent programme was given.—An enjoyable entertainment was given on Thursday evening last to the in-patients of the Cancer Hospital, Fulham Road, by Mr. Ernest Miles, a member of the honorary staff A hearty vote of thanks, proposed by Mr. C. H. Leaf, another member of the staff, was accorded with acclamation.

### Lectures to Nurses on Anatomy.

By W. JOHNSON SMITH, F.R.C.S., Principal Medical Officer, Seamens' Hospital, Greenwich.

### LECTURE VI.—THE SKULL.

IN studying the bones of the head it will be found very useful to have at hand a well-prepared adult skull, the upper part or vault of which can be removed to expose the interior. If possible the skull of the fœtus or infant should also be obtained for examination.

The skeleton of the head consists of a large integrate case of bone, the skull, and the loosely-attached lower jaw or *mandible.* The skull itself presents two well-defined segments; above and behind the large brain case, the *cranium or calvaria,* and, in front and below, the smaller part corresponding to the face. The cranial portion encloses and protects the brain, and together with the facial framework of bone includes four cavities occupied by as many organs of special sense: the organs of hearing, vision, smell, and taste.

The skull, though apparently a compact osseous box, is made up of not less than nineteen distinct bones. Most of

FIG. 13.—Upper Surface of skull.

A, Frontal Bone; B, Occipital Bone; C C Parietal Bones; D, Coronal Suture; E, Lambdoid Suture; F, Sagittal Suture; G. Bregma; H, Lambda.

these are separate in the infant, and in the fully-developed skull in which they are usually welded together their boundaries are well-defined by lines, some straight, others more or less intricate, which are called sutures (fig. 13). Of the nineteen bones, eight make up the cranium and eleven may be found in the face. In addition to these there are three bones in the cavity of the nose, and three bones, from their minute size called *ossicles,* in each internal ear.

*Upper Surface* (fig. 13).—The upper part of the skull called the *vertex* or the *cranial vault* presents a smooth convex surface of an elongated oval form, and broader behind than in front. This surface is traversed by one median and two transverse sutures which mark the boundaries of four flat or tabular bones: in front the single *frontal* bone, A, behind the single *occipital* bone, B, and between these bones, forming the top and sides of the vault, the two *parietal* bones, C. We should learn also the names of the sutures. That running across the top of the vault between the frontal bone and the two parietal bones is called the *coronal* suture, D (fig. 13), that behind, between the two parietals and the occipital bone is called the *lambdoid* suture, E; and, finally, the deeply serrated junction between the two parietals is called the *sagittal* suture, F. The point where this last suture strikes the coronal suture is called the *bregma,* G, and that behind, where its posterior end strikes the lambdoid suture is called the *lambda,* H.

The frontal bone which, as it is more or less prominent in different subjects, presents the high, flat, or receding forehead, forms at its lower part a considerable extent of the roof of the eye-socket or *orbit* on each side. In the cranial vault we see only a portion of the occipital bone which runs downwards and backwards, and finally forwards to form the back and almost one-half of the lower surface or base of the skull.

As we are considering the portion of the skull that is made up of so-called flat bones, it may be found convenient before passing to the other cranial surfaces to refer to certain structural details in these bones which will be found to present some points of interest and practical importance.

If we can obtain a prepared skull with a movable top, we shall find on detaching the upper part of the vault of the cranium that the thickness of the bony walls varies at different parts. The divided margin is very thick, in the middle of the section of the occipital bone, which indeed is the thickest part of the cranial vault, and less thick on either side of the skull. In the middle of the section of the frontal bone, although the space between the outer and inner surfaces of the bone may be increased, we shall probably find that some of this intervening space is occupied by portions of two cavities called the *frontal sinuses.*

The structure of the flat bones of the cranium is not uniform throughout the width of the surface of section. There is an external layer of compact and tough bone, an internal layer of compact bone which is said to be harder and more brittle than the external layer, and between the two a layer of "honeycombed" or "spongy" bone, which in a fresh skull is traversed by numerous blood vessels. The first layer is called the *outer table* (fig. 14), A, of the skull, the inner on account of its brittleness is called the *vitreous table,* B, and the intermediate layer of open bone structure is called the *diploe,* C.

Some of these anatomical data may be usefully applied in surgical work.

A good knowledge of the form and direction of the cranial sutures should prevent the error of mistaking one of these when exposed in a deep scalp wound, for a fracture of the skull.

In consequence of the cranial bones being thicker behind

FIG. 14.—Section of Cranial Bone showing A. Outer Table; B, Inner or Vitreous Table; and C, Diploe.

than at the sides of the skull, fracture of the skull is more likely to be met with in the latter than in the former region.

A fracture of the frontal bone just above the root of the nose, though deep, may not extend throughout the whole thickness of the bone.

In fracture of the cranial vault a slight depression or even a mere crack on the surface may be associated with extensive splintering of the brittle inner table.

The diploe with its numerous blood vessels presents in an open and severe head injury very favourable conditions for the reception and rapid breeding of the infective agents of inflammation.

### Beyond the Seas.

By an Occasional Correspondent.

### NURSING IN JAMAICA.

I noticed with interest an inquiry in The Hospital of November 9th for information concerning the hospitals and nursing homes of Jamaica. As I have just returned from a long visit to that island I am in a position to say something on the subject. Unfortunately, my experience is limited to the visits I paid these institutions; not having worked in any of them I cannot enter into the inwardness of their hospital life, but I shall do my best to give an accurate account of what I saw and heard, trusting that my trained eye took in more in one visit than an untrained eye would have done in a longer period.

### The Principal Hospital.

Kingston, the chief town in Jamaica, naturally possesses the largest public hospital. There are also 19 small hospitals in different parts of the island, with accommodation varying from 150 to 6 beds each, totalling 1,171 beds in all. But the number of beds fully equipped depends on the demand, and for the year ending March, 1900, did not exceed a daily average of 388 beds occupied, though the equipment had necessarily to be somewhat in excess of this. These institutions are visited by the medical officer of the district and are nursed by coloured women with, I should think, the most rudimentary knowledge of nursing. They are all supported by Government. The matron of the Kingston public hospital very kindly conducted me over the building on my first visit, and I am indebted to her for most of the information concerning it. It is a straggling building, very old, and in a shocking state of repair. Extensive alterations, in the way of enlargement and improvement, had been contemplated and plans made out, but owing to the financial depression all over the island they had to be abandoned. It is a great pity, for I believe as far as the nursing goes it has steadily improved. The wards, containing 200 beds, are divided into several separate blocks, none over two storeys high, which are all connected by covered ways to afford protection from rain as well as from the rays of a tropical sun. The theatre block, which is comparatively new, contains two surgical wards, male and female, each 72 feet by 22 feet; they open into the operation room, and have each a small room attached for critical cases. The theatre itself is an unpretentious little chamber with very primitive fittings, but I was assured by the matron that grave operations are performed there with most satisfactory results. The patients are nearly all of the poorest black and coloured classes of the community with a sprinkling of sailors from the vessels of all nations, with which Kingston harbour is crowded, excepting the British handy-man who has a magnificent hospital all to himself at Port Royal, on the other side of the Bay.

### The Nursing Staff.

The nursing staff consists of the matron, an assistant matron, six day and five night head nurses in charge of wards, four supernumerary nurses, and twenty-two under nurses. All the nurses are either black or coloured. The head and supernumerary nurses are selected from the better and more intelligent class of natives, who have received a fair education. The head nurses are in charge of the wards, the supers form a reserve force to take the place of the head nurse when absent, and assist in the wards when necessary. They wear a brown holland uniform, white cap and calico apron, the charge nurses wearing white collars and cuffs to distinguish them from the supers who wear turn-down collars and cuffs of the same material as their dresses. The pay of the head nurse varies from £1 to 14s. a week. The day or night duty is permanent and the hours are from 6 A.M. to 6 P.M. or *vice versâ*. The nurses are all non-resident, as there is no nursing home in connection with the hospital. The under nurses, who combine many of the duties of a wardmaid with the simpler duties of a nurse, are usually of a lower rank in life, but most of them can read and write. Women of this class are permitted to attend gratuitously and are trained in the duties of an under nurse; when sufficiently proficient they are expected to act in the place of an under nurse when necessary and then receive payment of from eight to nine shillings a week. Their uniform is a blue Galatea stripe, unbleached calico apron, and a Madras handkerchief tied round their heads. Though not entitled to a pension, it is usual when a nurse breaks down from ill health or old age after several years of service for her case to be taken into consideration and a moderate allowance, regulated by length of service, is granted. The uniform is provided by the hospital, and vacation leave is given as circumstances permit, four weeks being allowed during the year. Two of the female wards are under the supervision of a sister from the Mildmay Deaconesses' Nursing Home, who train nurses in these wards for private nursing in connection with the Home. They are generally coloured women of the better class and make excellent nurses. I believe that they are the only private nurses to be had in Kingston, or indeed in the whole island of Jamaica. Nursing is very rarely taken up as a profession by the white families of Jamaica. The climate is distinctly trying, and Creole girls as a rule have not much stamina.

### The Lying-in Hospital.

The Victoria Jubilee Lying-in Hospital forms a pleasing contrast to the ramshackle old public hospital. It is one of the prettiest buildings in the town, up to date in every respect, and as far as cleanliness and neatness go can hold its own with any hospital in England. The matron was away in England at the time of my visit, but her assistant treated me with every courtesy. The hospital was built entirely by small voluntary contributions from the people of Jamaica as a token of loyalty to the Queen, and is now supported by Government. It is a two-storeyed red brick building, with a wide open verandah along the sides, lighted by electricity, and has accommodation for 20 patients, the matron, assistant matron, and eight pupil nurses. The verandahs are covered with beautiful tropical flowering creepers as well as grape vines, which were literally loaded with magnificent bunches of grapes. The grounds are tastefully laid out in flower beds, and big tubs growing palms, and masses of maidenhair and gold and silver ferns in pots make the place a veritable Paradise. The wards are all in the upper storey, two large rooms containing six beds each, and six smaller rooms which are generally reserved for the married patients. Illegitimacy is, I regret to say, very prevalent in the West Indies, and the married women object strongly to share the wards with their less fortunate sisters The patients are all either black or coloured, and pay 10s. for the period they remain in hospital—generally 12 days. Should it be necessary for them to remain longer no extra charge is made. The beds were all full on the day of my visit, and the dear little fuzzy-headed black babies were on their best behaviour. I could not resist picking each infant up and giving it a cuddle, to the amusement of the assistant matron, who said that the lady visitors never troubled about the picaninnies – but then I do not suppose any of them were trained nurses, with L.O.S. certificates too, and the fact that the babies were black and brown, instead of pink and white, made no difference to me, they were so beautifully clean and quiet. The mothers were very proud of them. The labour ward was provided with two beds, and a table on which everything necessary for an ordinary accouchement was laid out, while a cupboard when opened disclosed a formidable array of midwifery instruments, antiseptic dressings, etc. I was told that there was seldom any occasion for using instruments, and that a black woman generally gets over her troubles very easily. We were next conducted over the ground floor, where the nurses' dining and bedrooms, lecture-room, and matron's charming sitting-room are. The nurses, all coloured, get from nine to twelve months' training. It used to be only six months, but this has very wisely been extended to nine or twelve according to their individual capability. Lectures are given three times a week by one of the public hospital staff, and the nurses are expected to take notes. Examinations are held at the end of the year by the lecturer, and certificates given which are signed by him as well as by the matron and the superintendent medical officer, who is the head of the medical department of the island. The kitchen, laundries, and store-rooms were subsequently inspected. The same immaculate cleanliness and order prevailed everywhere, though my visit was unexpected. The matron and her assistant deserve to be congratulated on the high degree of perfection they have attained in their hospital. I spent a most enjoyable afternoon going over it, and, on leaving, was given a lovely bunch of roses and lilies from the garden.

### Nursing Convalescents.

A Paper for Probationers.

"Nurse, will you please go to the matron's office at 11.15 to take some patients to the Convalescent Home?"

Perhaps some unpleasant moment of this sort is the first idea recalled to our minds by the word "convalescents." You remember the hasty rush from the ward to change into outdoor dress, the quick, concise directions given at the office, the four or five women and children sitting in a row in dejected attitudes, hoping, perhaps, that at least nurse's face may be a familiar one. Then comes the cab at the door and the beginning of difficulties: the stiff, heavy plaster leg that must rest on the opposite seat, the crutches that will not accommodate themselves except by leaning their heads out of window, the stout lady who cannot bear a breath of fresh air, the insufficiently-tied parcels into which inquisitive fingers have been poking until comb and brush, clean socks and handkerchiefs, part company with string and brown paper, and disappear from view; and unloading at the station brings another series of difficulties, until at last a friendly guard shuts the door on the whole party, and the much-harassed probationer heaves a thankful sigh, glad to be free to return to her beloved ward. Truly it is no pleasant business to carry a shrieking kicking child from the "sister" that it loves, and from all the other well-known faces, to the unknown and unloved "country"; for it generally happens that the child associates the country with the fearsome theatre that was the bourn of its last mysterious expedition.

It may be that a recollection of a different kind comes to us. One morning in the ward we found No. 3 bed filled by an unfamiliar face—drawn with pain and want of rest. Week by week he has been watched and tended, till at last—O happy moment!—the great authority allows No. 3 to get up for half an hour after tea. "Now mind, 3, no running about; just sit still here by the fire," says anxious nurse as she moves on to her next business. In five minutes she comes to have another look at him. Behold, an empty chair! while suspicious fumes from the bath-room lead nurse in that direction, to see, with horror, her cherished patient, leaning half out of the window, smoking a dirty clay pipe that has been smuggled away since last visiting day for this long-looked-for treat.

Well, there are drawbacks to the pleasure of nursing convalescents, but, at the same time, it is a real pleasure to see your patients struggling back to health, and there is always something to be learnt from them.

Take, for example, a convalescent typhoid boy. He has been promoted by easy stages from lying on a couch to being wheeled about in a chair, and then he is allowed to totter round the ward on a friendly arm, and to creep round his bed alone. In three or four days he finds his strength returning, and in his delight at being able to use his legs again he is on them all day long, running errands up and down the ward for the patients, following nurse on her rounds of bed-making or medicine giving till presently missing him, she hears dismal howls from the chimney corner, and finds her boy there, crying with pains in his legs. Real pain it is, and real tears roll down his cheeks. He declares that he can't stand, and finally she has to pick him up and carry him to bed, lucky if the pain is not too bad to keep him (and possibly his neighbours) awake till the middle of the night. And nurse has learnt that in some cases it is best to "make haste slowly."

There is another point about which to warn the inexperienced nurse who has the charge of a convalescent typhoid. These patients are apt to get sudden and inexplicable fits of temper: a small thing upsets them, and it is difficult to restore peace again. It is little good to coax, scold, or threaten the child; the nurse must remember that he is not yet well enough to be perhaps quite responsible for his actions, and she will be wise if she lets him have the disputed point his own way, or even shuts her eyes to his naughtiness for the time being.

I am not sure that adults are not affected in the same way; at any rate I remember a man being so much put out by not being allowed to get up the day that he considered himself well enough to make the attempt that he got out of bed and sat there, deaf to all arguments, giving no peace to doctor or nurse until a telegram was sent to his wife to come and take him home that very night.

It is a great advantage to be able to give convalescents a change of scene. In a private house they should be moved to another room for part of the day as soon as it can be managed, and allowed to sit in some sunny window to see a bit of outdoor life if they are not able to go out to get a breath of fresh air. Anything is good for a convalescent that takes him out of himself and helps him to get out of the way of thinking his own health the one important fact in the universe. And how the children love to sit on the window-sill, where they can see the happy street-boys playing cricket in quiet corners, or the fire-engines tearing past, or even just the fresh green leaves coming out in the spring! I shall not easily forget a little two-year-old boy jumping up in my arms when he was lifted up to the window one green June day to see the "Park." "Oh noice! oh noice!" the small voice shrieked in delight.

Finally, if it is not too trivial a detail, try to smarten up your patient when he begins to get well. Our hospital patients are by no means devoid of personal vanity, and a man will like himself better if he is wearing a smart dressing gown, with a bright-coloured rug wrapped round his knees, than if he is huddled up in a blanket taken off his bed. I need not mention the mental effect produced on women by the same means, nor yet the delighted admiration of the family on visiting days; and, after all, nice clothes are a part, though it is only a small one, of the civilising influences that we should try to bring to bear on our often rough patients.

### Lectures to Head Sisters.

By E. Margaret Fox, Matron of Tottenham Hospital, N.

LECTURE III.—A HEAD SISTER'S DUTIES TOWARDS HER PATIENTS.

The subject of your duties as head sisters of wards towards your patients is far too wide to be adequately treated within the limits of a single lecture. It reaches further even than your duties and responsibilities towards your probationers, and is far more important than your capabilities for good ward management. These are both necessary to the success of your hospital career, but this last will follow you outside the hospital, and make you, to every patient you nurse, ever afterwards either a comfort and blessing, or the reverse, according to your conception of your duties.

You may be excellent nurses, as far as knowledge of your practical work goes; you may understand exactly how to nurse any given case; you may know all about how to feed, wash, and clothe your patient; how to apply all the nursing remedies known to modern science; but if you lack that certain something—that indescribable gift of the gods that constitutes the "born nurse"—you will never be all you may be to your patients, and will find they often prefer others with less mechanical skill, but more sympathy, to wait on them.

The first duty—the duty which, if unfulfilled, makes all the rest barren and profitless—is then, undoubtedly, kindness, and the art of doing kind things kindly. This needs to be practised daily, hourly, in every dealing with your patients. It does not necessarily mean a great deal of talk—a sympathetic touch, look, or gesture often goes a great deal further than a stream of words, well meant though they may be: it means the taking of a little extra trouble to gratify a harmless whim, the going out of your way to do some little thing which, though not strictly your duty, yet makes all the difference between willing and half-hearted service—these are the things that endear a nurse to her patients, and which are remembered gratefully long afterwards. "The heart grows rich by giving," and in the very doing of countless little everyday kindnesses you have a sure and present reward.

Of course, however, you will never allow your kindly and sympathetic attentions to your patients to degenerate into spoiling them. Do not let them forget they are in a public institution where rules must be made and kept for the common good. Things must be done at certain fixed times, and discipline must be maintained; but if your patients get to know you always meet their wishes half way whenever you can, you will seldom have any real trouble with them when occasionally you insist on some course of action that is unpalatable to them. It certainly will not do to let them think they can behave exactly as they like in a hospital and have everything their own way. Spoiled patients are like spoiled children, fretful and unmanageable, a nuisance to themselves and everyone else.

It is important, too, to show no favouritism. To allow one patient some privilege not accorded to the others, for no better reason than that he or she happens to be a favourite, is distinctly wrong. Every nurse knows how hard it is to be just as bright and obliging to the grumpy, grumbling patient, always on the watch to find fault, as to the cheerful pleasant one, who will smile a "good morning" no matter how bad a night he has had, and who is ever hopeful about himself, and unwilling to give any extra trouble. It is not in human nature to like the one as well as another; but if you are going to manage your wards wisely and well, it is helpful to remember that all alike are ill and out of tune, and that all stand in equal need of your help and comfort.

Necessary it is, too, to guard against allowing any undue familiarity between the nursing staff and the patients. The sister of a ward should always keep herself on a higher level than the patients, and teach her probationers to do the same; not allowing any laughing or talking with them as with equals.

There is no objection to a convalescent's helping to wash up tea things in the ward kitchen, if you are alone on duty and the ward is busy: but he should not be there while the nurses are having a cup of afternoon tea, and he never ought to hear hospital affairs or the doctors and nursing staff discussed in his presence. It is well to cultivate a quiet, somewhat reserved manner with the patients, except in children's wards, where you cannot be too bright and cheerful with the little ones.

A great deal of the future conduct of your patients is determined by the kind of reception they receive on first entering your ward. Possibly they arrive at an inopportune moment. You may be just going round with the doctors, or giving out the dinners or going off duty; or you may be exceptionally busy, and have to think and plan how to fit in the new arrival. Take care that any of these circumstances, if present, do not tinge your manner with a certain coolness or brusqueness, that makes them wish most devoutly they had never come. However you may feel, it is your obvious duty to give newcomers a welcome, for often they are feeling very miserable indeed about submitting to be nursed at all by strangers.

Spare a few moments for a word or two, even if you are busy, and as soon as possible see about getting them comfortably in bed. Soften the ofttimes unwelcome fact of the regulation initial bath as much as possible, and get it done quickly, seeing that it gives no unnecessary discomfort. Chilly east winds through open windows, luke-warm water, needless prolonging of the process of drying, or waiting for things which should have been ready beforehand, all combine to make a bath a thing to be dreaded instead of the pleasure it ought to be.

In the interests of your patients, then, do teach your probationers to give a bath quickly as well as thoroughly. The two things are quite compatible, but it is quite surprising sometimes to see the leisurely, not to say dawdling way some nurses set about preparing a bath, and the length of time it takes to settle a new patient comfortably in bed. Consider their feelings too, when depriving them of a dirty vest or doubtful shirt, and console them by substituting a warm flannelette nightgown and bed jacket for the objectionable articles. Be sure and put on them at least as warm clothing as what you take off for there are few things that new patients more resent on your part, than the taking away of the soiled garments they are wearing. A want of tact in the matter will often make them very discontented.

*(To be continued.)*

### Presentations.

Hants County Asylum.—On Wednesday the 4th inst. the staff of the Royal Hants County Hospital presented a brass inkstand, letter-rack, and candlesticks to Mrs. A. K. Säch, who leaves Winchester this week to enter upon her new duties as matron of Malvern College Sanatorium. The presentation was made at a tea-party in Heathcote ward, and the visitors included patients from other wards, the nurses, some outside friends, and the domestic staff. The ward was very prettily decorated with yellow and white chrysanthemums. After tea the visitors gave a musical programme, and conjuring tricks were performed by one of the house doctors. Sister Säch will be greatly missed by the nursing staff, among whom she is very popular.

### Christmas Books.

### BOOKS FOR LITTLE CHILDREN.

The books published for children this Christmas by Thomas Nelson & Sons should have a great vogue with small folk. "Children of the Empire" is an illustrated rhyme book containing brightly coloured pictures of the children of our colonies. Commencing with youthful Australians the picture book concludes with a portrait of a small native of Jamaica. "A Day at the Zoo" will be a great favourite, being full of pictures of birds and beasts. "For the Flag," a painting book of the flags of all nations, will be a popular means of settling many a nursery dispute as to the nationality of the pennants displayed in the many processions we have seen of late. "Up to London to see the King" contains some delightful pictures of the principal sights of London and a beautiful panoramic picture of the metropolis as viewed from the Monument.

The Wood Pigeons and Mary. By Mrs. Molesworth. (Macmillan and Co., Limited.)

Like most of Mrs. Molesworth's stories, this little book abounds in graceful imagination. Mary is a little girl who lives in a London square, and makes friends and learns the language of two wood pigeons who built in the Square Gardens. How the pigeons migrated to the country, and were found again by Mary when on a visit to her godmother, is prettily told, and the pictures, by H. R. Millar, are worthy of the tale.

The Fish Crown Dispute. By Lancaster Lucas. (Skeffington and Son.)

This is a submarine fairy tale, telling of the adventures of two little children in a dream-world peopled by mermaids, fairies, and talking beasts and fishes. Mounted on the back of a stork the little brother and sister make a journey to Toy-land, passing Father Christmas' Castle on their way. The adventures are prettily illustrated in black and white by A. B. Woodward and F. B. Storey, and others.

The Lily Princess. By Margarite Floyd. (Skeffington and Son.)

The Lily Princess is all about three little children who, being thrown on their own resources for amusement, set out in search of a "Lily Princess," the heroine of a fairy tale read them by their nurse. How they found their Princess in the person of a dainty lady living in a beautiful house near by, the stories she told them, and the songs she sang them makes very good reading for small people.

Fairy Tales from the Swedish of Baron G. Djurklou. (William Heinemann.)

It would be hard to find a more acceptable gift book for a child than Baron Djurklou's delightful collection of Swedish folk and fairy tales. They were published nearly 20 years ago, but have now been for the first time translated into English by H. K. Brædstad. The illustrations have been beautifully executed by well-known Norwegian artists.

Queen Mab's Fairy Realm. (George Newnes, Limited.)

Fairy tales, culled from the literature of many lands, have been collected together within the pages of "Queen Mab's Fairy Realm." Not a few grown-ups retain a lurking taste for fairy stories, and this selection, accompanied as they are by exquisite illustrations, may fairly lay claim to a place on the drawing-room, as well as the schoolroom table.

The Olde Irishe Rimes of Brian O'Flynn. (Macmillan and Co., Limited.)

Though ostensibly a child's picture book these old "Rimes," so cleverly and humorously illustrated by Mrs. Praeger, will be even more keenly appreciated by grown-up people.

Old King Cole's Book of Nursery Rhymes. (Macmillan and Co., Limited.)

This is a very pretty rhyme book containing the old favourites—"Goosey Gander," etc.—with delightful coloured illustrations. It is a book to please old or young alike.

Bo-Peep. (Cassell and Co., Limited.)

A capital gift book for children. Lots of pictures and short stories and rhymes printed in large letterpress.

### BOOKS FOR BOYS.

Longfeather the Peacemaker. By Kirk Munroe. (G. Newnes, Limited.)

This is another tale of American Indians at the time of the Pilgrim Fathers, when the red man had been subjected to repeated outrages at the hands of the white invader. How a warm friendship sprang up between a great chief and Edward Winslow forms the theme for a narrative full of exciting adventure founded upon a historical basis. The principal incidents in the story have been illustrated in a spirited manner by Mr. Emlen McConnell.

Chapenga's White Man. By A. Werner. (Chatto and Windus.)

Chapenga was a shock-headed little native of Central Africa, who, after having been roughly treated by his own people, attached himself with unswerving loyalty and devotion to an English trader. How kindness and fair treatment turned the dross of his little impish character into, if not fine gold, at least some useful metal, makes very pleasant reading.

Jack Ralston. By Hampden Burnham. (T. Nelson and Sons.)

Jack Ralston was a young Canadian lad who went to seek his fortune with the Hudson's Bay Company. The story tells of how for five years he lived a life of exciting adventure and hairbreadth escapes from Indians. There is much about hunting and fishing in the book, which should prove a popular one with boys.

Acton's Feud. By Fred. Swainson. (G. Newnes, Limited.)

Another good book for boys is "Acton's Feud," a tale of public school life wherein the boys talk like real boys, quarrel, and make it up again like boys. The tale abounds in descriptions of football and cricket matches and in all that goes to make up English boy life.

The Boys' Odyssey. By Walter C. Perry. (Macmillan and Co., Limited.)

Mr. Perry has compiled an attractive boys' book, the "Odyssey of Homer," in narrative form, suited to the very young. Originally written for the amusement of his own boy, it will serve as a pleasant stepping-stone to the original for many a youthful reader.

God Save King Alfred. By Rev. E. Gilliat, M.A. (Macmillan and Co., Limited.)

A story about the great King of Wessex comes very appropriately this year, when we have just celebrated his Millenary. The tale abounds in stirring incident, and is beautifully illustrated by Gutzon Borglum.

The Heart of the Prairie. By John Mackie. (G. Newnes, Limited.)

Once more the "Great Lone Land" serves as the scene for a story of thrilling adventure. Cowboys, mounted policemen, Indians, and bears form the *dramatis personæ* of a capital boys' book.

Scouting for Buller. By Herbert Hagens. (T. Nelson and Sons.)

All boys embued with a love of soldiering and adventure should read this tale of the present war. It is told with knowledge and spirit, and greatly gains in interest by being historically correct.

### BOOKS FOR EVERYONE.

The End of the Epoch. By A. Lincoln Green. (W. Blackwood and Sons.)

This is by far the most convincing book of its kind that has yet been written. The idea of London being devastated and depopulated by some pestilence or other calamity is not wholly original, but "The End of the Epoch" may claim the great merit that, wherever the narrative touches upon scientific details, it is always absolutely correct. The story is based upon a highly imaginative idea—that of a terrible hybrid microbe being bred by an unscrupulous scientist, with the view of disposing of it to the highest bidder for destructive purposes. The scientist dies raving mad immediately after having discovered the antitoxin of the new germs. The only person inoculated with the antitoxin is a young scientific companion of the experimentalist, and he alone, with the exception of the very old, survives the pestilence, which is afterwards spread by the escaped germs, and tells the story. Many of the incidents are "creepy" beyond measure, and whether it will prove a book universally pleasing or not, it is safe to predict that few will put it down unfinished.

The Cankerworm. By G. Manville Fenn. (Chatto & Windus.)

A dramatic story, relating episodes in a woman's life. The opening chapters describe how an innocent schoolgirl was entrapped into a clandestine marriage by "the villain of the piece." The gay Lothario having given a false name, the marriage was illegal, and Linda, the heroine, found herself at seventeen deserted, penniless, and a mother. Time goes on; Linda, whose friends had come to her rescue allows herself to be married to an old Scotch nobleman who knows nothing of her sad story. How her child whom she believed to be dead, and her faithless lover, reappear on the scene of her life, is told with considerable dramatic skill. The story ends with the death of the villain by his own hand and the forgiveness of Linda by her deceived husband.

Dumb. By the Hon. Mrs. Forbes. (Chatto and Windus.)

Mrs. Forbes has accomplished some clever character drawing in "Dumb," which is a pathetic story of the experiences of a Scotch laird. First as a boy, and afterwards throughout his maturer life, Sir Alistair Craig bestowed his honest but undemonstrative affections upon an Irish maid, who, although she became his wife, never realised till too late how deeply he loved her. In Aileen, as child, wife, and widow, we have a delightful study of Irish character. Of course, "the other man" plays a part in the drama, but his is but a shadowy personality, and the interest of the book is focussed upon Alistair and his wife. It is a pretty tale but rather a sad one.

Valour for Victoria. By James A. Manson. (George Newnes, Limited.)

Twelve brave deeds that were rewarded by the gift of the Queen's Cross are recorded in this little book. The first British hero to receive the coveted prize was Midshipman Lucas, who, during the first bombardment of Bomarsund, at the commencement of the Crimean War, snatched up in his hands an explosive bomb hurled by the enemy and threw it overboard, thus saving his ship and all on board. The book closes with the pathetic story how Captain Towse, of the Gordon Highlanders, won his Cross and lost his sight during the heroic conflict with the Boers on Thoba Hill on May 5th, 1900.

Madamscourt. By H. May Poynter. (Thomas Nelson and Sons.)

The lives and loves of the Stuarts have provided novelists with an endless theme for romance. The account in "Madamscourt" of the escape of Princess Clementina Sobieski from Innsprück in 1719 to be married to Prince James Francis Stuart, is taken from a pamphlet entitled "Female Fortitude," published in London in 1722. The dates and historical facts of the narrative have been strictly adhered to, but the incidental personages who took part in the flight of the Princess and round whom the main interest of the story centres, are purely imaginary.

John Halifax Gentleman, by Mrs. Craik.

It is no easy task to choose a suitable book as a gift to our young girl friends, but for such a purpose it is always safe to turn to Messrs. Ward, Lock & Co.'s Lily Series. "John Halifax" now appears in that popular edition which is printed in nice clear type and provided with charming illustrations. Messrs. Ward, Lock & Co. have also brought out a new edition of "Invanhoe" by Sir Walter Scott. The Waverley novels are as eagerly read by some to-day as when they were first published, and this new edition, which is part of the "Youth's Library," is nicely bound and printed and contains the author's introductions and notes.

The House on the Scar. By Bertha Thomas. (Chatto & Windus.)

This is about a beach-comber who for a brief period relinquished his wild life of adventure, among the South Sea Islands and returned to respectable life in England, was received in Society, and wooed and won an English maid. Soon wearying, however, of the quiet life they led, Elliston returned to the old life, wrecking his young wife's happiness and finally losing his own life in a fight with natives. There is plenty of dramatic incident throughout the book and the interest is well sustained to the end.

Diseases of the Cat. By J. Woodroffe Hill. (Baillière, Tindall and Cox.)

Many people interested in the feline race will welcome this little manual, the result of Mr. Woodroffe's many years of close research into the maladies of the cat. The book contains several prescriptions and antidotes for poisons which should be invaluable in rendering "first aid" before veterinary assistance can be obtained. Some charming portraits of prize cats adorn the pages and the cover.

Britannia's Bulwarks. Edited by Commander Robinson, R.N. (G. Newnes, Limited.)

The achievements of our seamen and the honours of our ships are in this book presented by picture and pen. A story of prowess is told by the hand of a writer well versed in the history of our Navy, while the series of water-colour pictures by Mr. Charles Dixon, R.I., and the monochrome drawings by Mr. S. Staniland, R.I., add greatly to the value of the work.

Uncle Tom's Cabin, by Harriet Beecher Stowe.

Messrs. Ward, Lock & Co. have added "Uncle Tom's Cabin" to their Prize Library edition. Although the noble purpose which inspired the book has long since been accomplished, "Uncle Tom's Cabin" continues to be read with keen interest. This new edition, like the rest of the Prize Library, is attractively bound and furnished with a few excellent illustrations.

A Blind Marriage and other Stories. By George R. Sims. (Chatto & Windus.)

The first of these stories tells of how a man lost his sight and married the wrong sister. All ends happily as indeed do the other tales in the volume. Most of them are Christmas stories told in the bright and cheery style familiar to us from the pen of Mr. Sims.

The Twentieth Century Citizen's Atlas. Edited by J. G. Bartholomew, F.R.G.S. (G. Newnes, Limited.)

This very useful atlas is being published fortnightly in 26 parts. Commercial charts are a special feature of the publication which contains 156 pages of maps and plans, with an index, a gazetteer and geographical statistics.

Ugly: a Hospital Dog. Recitations and Readings by George Dabbs, M.D. (Charles William Deacon and Co.)

Ugly is made to tell his own stories, and very amusing they will prove to dog lovers. Besides the hospital bulldog's autobiographical recollections are some recitations and readings in prose and rhyme for odd hours.

The George, Prince of Wales, Prayer Book.

An acceptable present for a friend this Christmas-tide would be a "George, P ince of Wales," Prayer-book. The new edition of our Common Prayer, which the Oxford University Press have just published, is of convenient size and tastefully bound in morocco with gilt edges and gold lettering.

The Animals of the Bible. By Gambier Bolton, F.Z.S.

The Bible is full of references to the creatures inhabiting Palestine, Assyria. and Egypt. Mr. Gambier Bolton has given an account of all these in his little book which is illustrated from photographs taken by himself at the Zoological Gardens.

CHRISTMAS NUMBERS OF MAGAZINES.

There is much good reading for quiet Sundays in this year's *Sunday Magazine.* The long serial, "The Winds of Cathay," by Christabel Coleridge, is full of interest, and the illustrations are plentiful and well executed. Amongst the more serious contributions is a series of interesting biographical sketches by F. B. How, entitled "Noble Women of our Time" The Christmas number of *Cassell's Magazine* contains the opening chapters of a new story by W. Le Queux, and a variety of short stories and illustrated articles by well-known writers. Every number contains five Rembrandt photogravures, and a large plate copy suitable for framing of Mr. F. Gribble's great picture "The Pirate's Prize," is presented to each purchaser. The Christmas pnblication of the *Boy's Own Paper* is full of bright stories and articles on subjects interesting to boys. As a frontispiece there is a double-page coloured plate representing a football scrimmage. The extra Christmas part of the *Girl's Own Paper* is attractive within and without. "Love's Sacrifice" is the name of a pretty story in 25 chapters. The rest of the pages are devoted to a few short stories and articles on cooking, etc, interesting to the modern maid. The *Gentleman's Annual* contains this year a complete novel entitled "As it was Written," by T. W. Speight. The story turns upon the homicidal instincts of a Lady Gwendolen Harlett, and is full of thrilling incident. The publication of *Pears' Annual* is accompanied this Christmas by three large coloured plates, "Witchery," taken from a painting by A. Piot; "The Coming Nelson," from the original painting by Fred Morgan; and "Little Bobs," from the picture by Edgar Bundy. Among the contributors to the Christmas number of the *Windsor Magazine* are Hall Caine, Rudyard Kipling, Guy Boothby, and other popular writers. The illustrations are unusually good, and include two by Maurice Griffenhager, and several by Raven Hill The *Captain* is a great favourite with lads, and the bound volume of the last half-year's issues seems to contain matter on every subject likely to interest boy readers. There are some capital stories, both long and short, and the illustrations are excellent.

Little Folks. (Cassell and Co., Limited.)

Despite the many magazines which wax and wane, "Little Folks" maintains its reputation as one of the first favourites in children's books. The stories and pictures in this year's issue are as good as those which delighted our own childhood. The Christmas number for 1901 is brightly bound, and has a nice coloured frontispiece.

Tiny Tots: a Magazine for very Little Folks. (Cassell and Co., Limited.)

We might search far before finding a more welcome gift for a small person than "Tiny Tots." Nonsense rhymes and funny stories and pictures make up a volume that may claim to meet even the imperious demands of the modern nursery.

### Christmas Novelties.

By our Shopping Correspondent.

NURSES' APRONS.

Messrs. Hanna, of Belfast, who manufacture all sorts of linen goods, have a special apron for nurses, which should prove both strong and durable. It is made of extra heavy Irish linen, and may be had plain or with hem-stitched edges, according to requirements. I advise nurses to send for patterns.

THE BEST EAU DE COLOGNE.

Can I say any good thing that has not already been said of Mühlen's 4711 Eau de Cologne? It is an ever acceptable present, and one that is most appropriate, above all, for an invalid. There are properties in this Eau de Cologne which help to render the handkerchief antiseptic, so that it is not only delightful as a perfume, but useful also. Nearly every shop that sells perfume nowadays sells 4711 Eau de Cologne, but in case any difficulty in procuring it arises, I may mention that the English depôt is 62 New Bond Street, where Messrs. Mühlens sell also all kinds of tempting scents and accessories of the toilette.

DIARIES.

Nurses who are looking out for dainty gifts for friends should visit the showrooms of Messrs. Jon. Harris & Sons, Ltd. 25 Old Bond Street. Here they will find a variety of pretty things—diaries and calendars for 1902, bound in Harris art linens, and worked in various colours, engagement cards, work-bags, "early-morning trays" (with embroidered tray cloth), handkerchief sachets, etc. The "week-end sachet" should appeal to those who are always on the wing; it contains all necessaries for a night or so, and is very prettily worked. For those who have time to embroider their own gifts, I may add that all the worked articles can be had traced and begun, while nurses with a taste for making their own designs will find a varied choice of colour in linens and flax threads, and may pick up many new ideas as well in the course of a visit. All the materials are manufactured at the Derwent Mills, Cockermouth, specially for Messrs. Harris.

### Our Christmas Supplement.

With the present issue of The Hospital is presented an illustrated supplement, "Hospitals Within the Empire," being a series of articles describing Christmas in the hospitals beyond the seas, written by matrons, sisters, and staff nurses.

### To Nurses.

**We invite contributions from any of our readers, and shall be glad to pay for "Notes on News from the Nursing World," or for articles describing nursing experiences, or dealing with any nursing question from an original point of view. The minimum payment for contributions is 5s., but we welcome interesting contributions of a column, or a page, in length. It may be added that notices of appointments, entertainments, presentations, and deaths are not paid for, but that we are always glad to receive them. All rejected manuscripts are returned in due course, and all payments for manuscripts used are made as early as possible after the beginning of each quarter.**

### Help the Nurses to help the Sick.

**Royal National Pension Fund for Nurses,** 28 Finsbury Pavement, E.C.—This fund has during the past year more than maintained the remarkable and uninterrupted success which it has enjoyed ever since its establishment. The number of policies issued in 1901 was over 800, or more than 100 in excess of 1900. Upwards of £1,600 has been distributed in sick pay to members of the Pension Fund; a fact which cannot fail to particularly impress nurses who are working on their own account. About £5,750 was paid away in pensions and bonuses in 1901, an increase over the previous year of no less than £1,200. The premium income, *i.e.*, payment by, or for, nurses exceeded £83,000, the total income for the year being near £110 000. The invested funds of the society now stands at £640,000.

**The Junius S. Morgan Benevolent Fund** is an auxiliary to the Royal National Pension Fund for Nurses, and was founded through generous contributions from nurses themselves, and raised to handsome proportions by the munificence of the Morgan family and many other friends to nurses. The work is done by volunteers, under the supervision of an influential committee, which devotes time and care to the investigation of claims and the relief of urgent cases of distress amongst the policy-holders in the Pension Fund. Hon. Secretary, Miss Rosalind Pritchard.

**"The Hospital" Convalescent Fund.**—The object of this fund is to provide rest for weary workers amidst suitable surroundings, without any of that anxiety about ways and means which retards convalescence. Since the establishment of it many tired and delicate workers have enjoyed a much-needed change of air such as they could not possibly have secured for themselves without help. Experience has proved that it is better to let the nurses have a choice of locality rather than to send them to one settled place, and nurses are accordingly sent to all parts of the country. Contributions which would increase the field of usefulness are invited by the Hon. Secretaries, care of the Editor of The Hospital.

**Queen Victoria's Jubilee Institute for Nurses.** Offices: St. Katharine's Precincts, Gloucester Gate, Regent's Park, N.W.—The Institute trains nurses in district nursing, and supplies nurses to affiliated associations for the sick poor in their own homes. Applications for information should be addressed to Miss Peter, the General Superintendent. Nursing associations in England, Scotland, Ireland, and Wales are affiliated with the Institute.

**East London Nursing Society.**—The object of this society is to nurse the sick poor in East London in their own homes by means of trained resident nurses, each nurse living in the parish in which she works. The extent of the society's useful work is shown by the fact that in 1900 the staff of 30 nurses attended to 5,028 persons, to whom 109,037 visits were made. Annual subscriptions and donations to the general fund are asked for. Secretary, Mr. Arthur W. Lacey, 43 Rutland Street, New Road, Commercial Road East, E.

**The Colonial Nursing Association,** the Imperial Institute, S.W.—This valuable association was founded five years ago to supply trained nurses to the Crown Colonies and small British communities in foreign countries. Since the foundation 104 nurses have been despatched to various parts of the world, grants in aid being made where it is clearly shown to be impossible for the residents unassisted to bear entire cost of passage moneys, salaries, and maintenance. It is one that appeals to the sympathies of all, for what family is there that has not some members in distant lands, building up the Empire, and fighting with the sickness that comes with rough faring and undrained country? The Hon. Secretary, Mrs. Debenham, will be glad to receive contributions, especially as an effort is being made just now to extend its benefits to the poorer colonies.

### For Reading to the Sick.

VERY PITIFUL AND OF TENDER MERCY.

O foolish human heart that wrongest Me,
How long shall I bear with you, yea, how long
Suffer you still to take My Name in vain?
How can those half-blind eyes that scan the gloom
See anything aright of all My work,
And, seeing not, why judge Me in the dark?
Perchance some day the clearer light will show
That pain, disease, and grief are gifts as great
As strength and health and joy, which seem so dear.

*F. M. Owen.*

---

You watch a life bereft of light,
For ever wrapt in unthinned gloom,
Whose only tranquil time seems night,
Whose happiest hope and rest the tomb:
I watch the life, and know that God
So guides the soul to Heaven above,
You only see the smiting rod—
But, ah! the Power that smites is love.

*A. Norris.*

---

Very pitiful and of tender mercy. So he ever moves among His fellow men; He, the sinless and almighty. No gentle and sensitive woman ever drew near to suffering sorrow with a pity so delicate and entire as His. No misery seems so remote from the outward circumstances of His life, no anguish is so well deserved that he can pass it by: there seems no limit and no denial in the generosity of His compassion; and from his presence, even in all the strength and majesty of perfect holiness there ever streams a grace and radiance of pity at which the most secret sorrows of the world are disclosed and turn to him as flowers in the sunlight.

God puts within our reach the power of helpfulness, the ministry of pity: He is ever ready to increase His grace in our hearts, that as we live and act among all the sorrows of the world we may learn by slow degrees the skill and mystery of consolation: not only has He had pity on us, but He also suffers us to know the blessing and the happiness of entering, with the gentleness of a pity not utterly unlike his own (just because it is indeed His gift), into the troubles and the wants of others. "If you know these things, happy are ye if ye do them." There is no surer way of steadfast peace in this world than the active exercise of pity; no happier temper of mind and work than the lowly watching to see if we can lessen any misery that is about us: nor is there any better way of growth in faith and love.—*Extracts from "The Inheritance of the Saints."*

---

When joy no longer soothes or cheers,
And even the hope that threw
A moment's sparkle o'er our tears,
Is dimm'd and vanished too!
Oh who would bear life's stormy doom,
Did not Thy wing of love
Come, brightly wafting through the gloom,
Our peace-branch from above?
Then sorrow, touch'd by Thee, grows bright,
With more than rapture's ray;
As darkness shows us worlds of light
We never saw by day.—*T. Moore.*

### Everybody's Opinion.

[Correspondence on all subjects is invited, but we cannot in any way be responsible for the opinions expressed by our correspondents. No communication can be entertained if the name and address of the correspondent are not given as a guarantee of good faith, but not necessarily for publication. All correspondents should write on one side of the paper only.]

### THE NURSE'S NEVER.

"A NURSE" writes: I am rather astonished at the tone of some of the comments written by nurses, on "The Nurse's Never." Why should we be so irate at such plain and excellent advice? Naturally it is not meant for perfect nurses, nor need they take it as given to them, but we are not all perfect. As I read not long ago in THE HOSPITAL, "the average nurse is the average woman;" so there must be many of us who are not harmed by a timely warning. It is so easy to turn this sort of thing into ridicule and take up the position that it is impossible for a nurse to make mistakes. In my own personal experience which has not extended over many years, I have known accidents happen to well-trained and conscientious nurses from the neglect of some of these "Nevers." Would it not be more becoming in us as nurses who love our work, to be grateful for all good advice whether we need it or not? and say, "Thank you very much."

### WHY ARE NIGHT NURSES NEGLECTED?

"LANCASHIRE" writes: Unlike your correspondents of last week I am in sympathy with "Yorkshire" upon the deterioration of nurses. Nowadays they often think too much of self, and fail to place their work first as they should do, to be thorough. In my opinion the public are largely to blame for the exalted opinion so many nurses hold of themselves. Their work is lauded and their lives are described as of such a noble and self-sacrificing nature, that it can hardly be wondered that they become captious and exacting. Why should nurses expect so much more consideration than other ranks of educated workers? The work is said to be trying, but surely the many pleasures we can derive from our work, well and faithfully done, will always counterbalance the vexations which are inseparable from our daily life, let our occupation be what it may. It has always been my conviction that a night nurse's diet requires more consideration than that of others, but whatever is done there will be grumbling, which appears to increase as time advances. If I found all my nurses contented I should wonder what could have happened.

### SCARCITY OF NURSES IN WORKHOUSES.

"ANOTHER WORKHOUSE NURSE" writes: Referring to the letter under this heading in your issue of December 7th, surely "A Poor Law Guardian" is rather hard on a Workhouse nurse. His Union may boast one of the good types of master and matron, of which there are many, but he should look at both sides of the question. A nurse resents interference with her duties as much as, for instance, the master would resent the nurse admitting or discharging inmates, meddling with his books, etc. I repeat a Workhouse nurse's question, "What can Guardians expect if they allow untrained, uneducated matrons (and masters I include) to interfere with a nurse's duties?" Why have trained nurses at all, if untrained superior officers are set over them with the power to interfere? Then again, where do the guardians gain their knowledge of what a nurse's duties are? Surely all or any interference should emanate from the doctor whose province it is to manage the nursing staff. Whatever may be the faults of a nurse (and I do not pretend to argue that she is flawless) at least it must be admitted that her main object is to alleviate the sufferings of the poor unfortunate beings under her care, and as a rule a nurse has not only learnt her work but also the lessons of patience and kindliness. I know from sad experience that the requisites for the support and comfort of the sick paupers is the chief bone of contention between master and nurse, or matron and nurse. The latter usually leans to the side of kindness; the master seemingly thinks his dignity is lowered by the mere act of being kind to paupers, or he wishes to stand well with the guardians by keeping the expenses down. He may be the best-natured creature in the world at heart, but he invariably goes very much out of his way to conceal this weakness. It would never do for paupers to be pampered. Judging from a personal knowledge of the inner workings of both workhouse and infirmary, it seems to me that the root of the evil lies in the fact that there is no superior authority to weigh the correctness of the nurse's demands and the master or matron's refusals. The master is counsel, judge, and jury combined in himself, consequently it is not surprising to find the verdict frequently in his own favour, whilst the Guardians, regarded as a court of appeal, will generally be found to uphold the judgment of the lower court. As your correspondent rightly says, "many of the Poor Law Guardians have occupied their seats for 20 and even 30 years on their various boards." This is precisely the cause of complaint against them, that they do occupy their seats with masterly inactivity. Let the Poor-law Guardian investigate for himself the facts. Let him, or those he can trust, become paupers *pro tem.*, so that they may see the working behind the scenes as well as from the stalls and discover where the fault lies. Then, probably, the scales will fall from his eyes and he will find that the scarcity of Workhouse nurses is not without reason. The title Poor-law Guardian is an honourable one, and the men who hold office are usually men of honour. But do these honourable gentlemen fully realise their responsibility for the welfare of their fellow creatures, which in reality rests upon them? I ask the question with all due respect.

### Appointments.

BOROUGH HOSPITAL WOLVERHAMPTON.—Miss Charlotte Wynn has been appointed staff nurse. She was trained at the Women's Hospital, Liverpool, for three years, and has since been for four years staff nurse at the General Hospital, Spalding. She has also done private nursing.

CROYDON INFIRMARY.—Miss Bertha Connolley has been appointed sister. She was trained at the Walsall Infirmary. She has since been charge nurse and midwife at the Whiston Infirmary, Prescot. She holds the L.O.S. certificate.

NO. 5 STATIONARY HOSPITAL, BLOEMFONTEIN.—Miss Ridley Makepeace, Army Nursing Sister, who for nearly two years has been acting-superintendent of the hospital-ship *Avoca*, has been appointed superintendent.

PADDINGTON INFIRMARY.—Miss Maude Bateson has been appointed night superintendent. She was trained at Mill Road Infirmary, Liverpool, where she remained as ward sister for three years. For the last fourteen months she has held the post of sister at the Wimbledon Isolation Hospital.

ROYAL HOSPITAL, PORTSMOUTH.—Miss Hettie Shorto has been appointed assistant matron. She was trained at the Royal Surrey County Hospital, Guildford. She has since been staff nurse and private nurse at the Sussex County Hospital, Brighton, and for the last three years sister of the male surgical and accident ward, Royal Hospital, Portsmouth.

ST. PANCRAS INFIRMARY, COOK'S TERRACE, N.W.—Miss Mary Thompson has been appointed night sister. She was trained at St. Saviour's Infirmary, East Dulwich, was ward sister at the Poplar and Stepney Sick Asylum for two years, did private and hospital work in Algiers for eight months, and for the last year has been ward sister at the St. Pancras Infirmary.

SWANSEA UNION WORKHOUSE INFIRMARY.—Miss Nellie Stewart and Miss Frances Broadhead have been appointed charge nurses. Miss Stewart was trained at St. Pancras Infirmary, and has since been charge nurse at Portsmouth Infirmary. Miss Broadhead was trained at Manchester Royal Infirmary, and has since been charge nurse at Whiston Infirmary, Prescott.

### Echoes from the Outside World.

**AN OPEN LETTER TO A HOSPITAL NURSE.**

NURSES who wish to make their arrangements for next year to allow of their being present at some of the functions in connection with the Coronation, will like to know that the King has fixed Thursday, June 26th, as the date of the ceremonial itself. But probably the following day, the 27th, will be the one upon which most of his subjects will have a chance of showing their loyalty, as it is proposed that the route along which the King and Queen and their visitors will drive that day shall be an extended one. The question of the flower to be worn upon Coronation Day is still occupying public attention, and market gardeners in particular are most anxious that some decision shall before long be arrived at, so as to give them a chance of growing the chosen blossoms in sufficient number to meet the enormous demand. Those who think that the lily of the valley should be selected are told that though it would be a pretty compliment to the Queen, it is not enough of a general favourite for its choice to be popular, and a plea has been put forth for the carnation. The name of the flower means "Coronation," being the old name given to it by the poet Spenser and others, and it is easy to obtain in the latter end of the month of June. But the loudest and strongest demand is for the rose, the flower of England, the token alike of Yorkists and Lancastrians, and the flower to be found as easily in the garden of the tiny cottage as on the parterre of the palace or the mansion. The only objection which can be urged to the choice of the rose is that it is already worn on St. George's Day. Of course it is, but St. George is our patron saint, and his flower should be especially in evidence on June 26th.

THOSE who followed closely the Colonial tour of the Duke and Duchess of Cornwall must have been impressed with the universal excellence of the addresses delivered by the Heir Apparent. But the majority of busy English people do not devote much time to matters which they do not think nearly concern them, and the speech made last Thursday by the Prince of Wales in the City came as somewhat of a revelation to many. Indeed, the country has suddenly awakened to the knowledge that His Royal Highness is an orator endowed with talents of no mean order. When Lord Rosebery—himself a fine speaker—characterised the address as "eloquent and statesmanlike," he but echoed the judgment of all who heard the words at the Guildhall and of all who subsequently read them. The clever manner in which the Prince gently led his hearers from place to place, carefully mentioning all the countries which had given him such hearty welcomes, paying to each some honest tribute which had about it the ring of sincerity, till he arrived at Newfoundland, our oldest colony, was particularly happy. And his concluding words were especially noteworthy. Of the features of the tour which had most impressed him, he unhesitatingly placed before all others the devotion to the Crown and the attachment to the Old Country, and expressed his firm belief that it was the life and example of our late beloved Sovereign which had fostered this spirit of loyalty and love. Then came a word of warning, suggested by what he had heard and by the signs of progress he had noticed everywhere beyond the seas. The old country, he said, must wake up if she intended to maintain her old position of pre-eminence in her colonial trade against foreigners. The great need of the Colonies everywhere was increased population, and the Prince appealed to his fellow-countrymen at home to prove the strength of the attachment of the motherland to her children by sending to them "only of her best." Since the speech at the Guildhall was made, it has transpired that the Prince of Wales never leaves the composition of any of his addresses to any member of his staff, but prepares them himself, even down to the smallest detail. This means conclusively that his words reflect, not the sentiments of others, but his own convictions.

IN one of Gilbert and Sullivan's comic operas there is a song which has as its refrain, "A policeman's life is not a happy one." The same sentiment might, it seems to me, be applied with even greater truth at the present time to the king or queen of almost any country in any clime. Of late it is Queen Wilhelmina, who has been realising, with much bitterness, that personages in royal circles are not even allowed the privilege of "a few words," as it is termed, without the whole of the civilised world ringing with the news of "a serious quarrel," "a prince insulting a queen," "two duels," "sad illness of the insulted queen," "impending separation," etc. I am sure that all who have read the recent reports from Holland can only deeply regret that the private affairs of the royal pair should have been thus discussed by an ill-restrained press. Perhaps Prince Henry and his wife were not quite of one mind upon some point. Are they the only couple who have failed to get through the first year of married life without a jar? Why should such a disagreement be published at once with every possible embellishment for the edification of the general public? Because an aide-de-camp has peritonitis is it of necessity the result of a duel fought by him to defend the cause of his Queen against her own husband? An emphatic denial has been given to the details supplied to some of the daily journals, but unfortunately it is so easy to make mischief and so difficult to undo it. However devoted Prince Henry may prove himself to be in the future, and however loving and sweet Queen Wilhelmina may be to her spouse, there are some miserable folks who will never forget that in the early years of their marriage they once had, or were supposed to have had, a quarrel, which, instead of being quietly made up in the sacredness of home was blazoned abroad for the sake of providing sensational copy and attractive headlines.

ALTHOUGH there is nothing new under the sun, articles which have not been seen for so long that they may be classed as novelties are constantly being brought forward, especially just now. Amongst the nicest gifts for women are fur bags to match boas, muffs, and coats. They are about the same size as the little black satin bags, or those made of the same material as the dress, which are so much patronised by the poor pocketless woman of the present era, and are meant to contain a tiny purse and a pockethandkerchief. The more expensive specimens somewhat resemble the old-fashioned reticule, and are fixed on to chased gold frames with chains to attach them to the waistband, or, if preferred, they may be slipped over the wrist. But those who have clever fingers can make a very good copy of the fashionable bag for a small cost, and of course it is only a passing mode. Perhaps a friend has a nice black astrakan collarette and muff Fashion a pretty-shaped little bag, cover it with a piece of fur—half a yard of a wide width would answer the purpose —line the inside with black satin, edge with a frill of the same or with black silk cord, ornament the top in like manner, and behold a neat little bag for a Christmas gift, which can always be used when the furs are worn. The same idea can be carried out with any other fur, but in the case of light furs, satin or cord to match is not pretty, and it is better to make it up with velvet of a darker tone or with the same coloured cloth as the costume which the future recipient usually wears.

### Notes and Queries.

**The Editor is always willing to answer in this column, without any fee, all reasonable questions, as soon as possible.**

**But the following rules must be carefully observed:—**

1. **Every communication must be accompanied by the name and address of the writer.**
2. **The question must always bear upon nursing, directly or indirectly.**

**If an answer is required by letter a fee of half-a-crown must be enclosed with the note containing the inquiry.**

*Waiting Fee.*

(101) I am a maternity nurse, and have had to give up a case in order to be ready to attend the lady who engaged me at the beginning of the month. Can I charge for the waiting time, and what would be a reasonable fee?—*Monthly Nurse.*

You are entitled to your fee from the date of engagement, not before. An arrangement is sometimes made to pay the nurse half fees from the date of engagement until that on which her services are required.

*Supplementary Training.*

(102) Do you know of any hospital where a partially-trained nurse could be received at a lower fee than that usually required for special probationers, in consideration of her previous training? *B. M. S.*

Matrons have a natural objection to partially-trained probationers.

*Norwich.*

(103) Can you tell me if there is a Nurses' Co-operation in Norwich?—*M. A. B.*

The Norfolk and Norwich Staff of Hospital-trained Nurses, 50 Bethel Street, Norwich, appears to be the only institution for nurses in that city managed by a committee; and its terms are not arranged on co-operative principles.

*Badges.*

(104) Can you kindly tell me of a good firm where nurses' badges are made?—*H. N. I.*

Any firm catering for nurses' specialities would procure them. See our advertisement columns.

*Lysol.*

(105) Will any midwife tell me if she uses "Lysol" in her work; and if she finds that it causes a rise in the patients' temperature. *Worried.*

Perhaps you use too strong a solution. 1 per cent. is the strength commonly used.

*Nursing Charities.*

(106) Would you kindly inform me for what charities a trained nurse, elderly, would be eligible? She has no income when not working.—*M. S.*

The usual charities for ladies, the Trained Nurses' Annuity Fund, 72 Cheapside, E.C., and possibly the Junius S. Morgan Benevolent Fund, 28 Finsbury Pavement, E.C.

*Ambulance Classes.*

(107) I am anxious to attend some ambulance classes in Liverpool; will you kindly tell me when and where the lectures are held?—*F. T.*

Apply to the Secretary, St. John's Ambulance Association, St. John's Gate, Clerkenwell, E.C., and ask what arrangements have been made for local lectures in Liverpool.

*Home Nursing.*

(108) Can you tell me what more is required than the training of a nurse to take up lecturing in Home Nursing?—*Work.*

The County Council lecturers must be certificated teachers of their subject.

*Nursing in America.*

(109) 1. I want to enter a hospital in Chicago, U.S.A., as probationer. Can you give me the names of some to which I could apply? 2. Is there a paper corresponding to The Hospital in the United States?—*Chicago.*

You will find a list of the chief training schools for nurses in America in "The Nursing Profession: How and Where to Train." 2. Not exactly. *The Trained Nurse* is one of the best publications there.

1. Can you tell me if a three years' certificate of general training, and also one of midwifery obtained in England, would be recognised in America? 2. Is there a three months' midwifery course to be had in America, as in England, costing from £10 to £25?—*M. H.*

1. Yes; but English nurses have to combat many difficulties. 2. No; trained nurses can receive six months' instruction in maternity nursing at Boston Lying-in Hospital, M'Lean Street.

*Untrained.*

(110) I am not trained, but I have had a great deal of private experience in nursing. I want to obtain a post as nurse-companion, but, although I have answered several advertisements, I have had no reply. Would you advise me to advertise?—*J. H.*

Yes.

*Visiting Nurse.*

(111) Whilst staying in a country village, I find from the medical man that there is great need of a visiting nurse. The inhabitants are working people, earning from 20s. to 25s. a week. What charges would you advise me to make?—*Nurse E. S.*

We cannot advise you, as you must find out what you can live on, and what your patients can pay. Could not your medical man form a committee from amongst the inhabitants, and guarantee you a salary? There must be a certain amount of charitable work done under such circumstances, and the nurse must live.

Will you kindly tell me what would be the usual scale of charges for a visiting nurse to make in a provincial town?—*Kiaraw.*

From 2s. 6d. to 3s. 6d. per visit, making reductions for regular attendance. Operations, etc., from £1 1s. All is, however, regulated by the means of the patient, and the length of the visit.

*Maternity Nursing.*

(112) I am about to study midwifery, and should be much obliged if you would kindly tell me which would be the best book on that subject for me to begin with?—*Dishie.*

A "Practical Handbook on Midwifery," by Francis W. N. Haultain, M.D. Price 6s.

Is it a much greater advantage for a nurse going in for private maternity work, to take midwifery rather than only monthly training?—*Blanche.*

Certainly, and, if possible, she should take general training as well.

Will you kindly tell me if there is any midwifery training school which would give free training in midwifery to a fully-trained hospital nurse?—*A. A. D.*

See advertisements in The Hospital *Nursing Section*, or advertise.

1. How should one proceed to become a maternity nurse? Is there a hospital near Liverpool. 2. Is the examination difficult?—*Ellen.*

You should give your surname for private information. 1. Liverpool Ladies' Charity and Lying-in Hospital, Brownlow Hill. Resident pupils, £18 18s.; non-resident, £7 7s. The Mill Road Infirmary. The Workhouse Infirmary train their probationers in maternity. 2. Not to an intelligent, diligent student.

Having been left through death quite unprovided for, I am desirous of obtaining a certificate in maternity nursing. Can you tell me how I can possibly get into some hospital as I am over the age usually admitted?—*Card Enclosed.*

The card has unfortunately been mislaid. Would our correspondents kindly always write their names and addresses on their letters. Advertise, or take lessons in a private home. See reply to *A. A. D.*

*Army Nursing Reserve Service.*

(113) Will you tell me how to join the Army Nursing Reserve Service, and, if I were accepted, should I have to wait until I was wanted, or could I volunteer for immediate service?—*Slade.*

Apply the Hon. Secretary, 18 Victoria Street, S.W. You can volunteer for immediate service.

Would you kindly tell me if a nurse with a three years' certificate from a large union infirmary is eligible for Army nursing and able to join the Army Nursing Reserve?—*Nurse Dora.*

Yes. See reply to *Slade.*

*Sanitary Inspector.*

(114) Please tell us what steps a certificated nurse should take in order to become qualified as a sanitary inspector.—*D. C.* and *Nurse S.*

Apply to the Sanitary Inspectors' Examination Board, 1 Adelaide Buildings, London Bridge, E.C.; or to the Sanitary Institute, Margaret Street, W., and ask for particulars of the examinations, which you must pass in order to be qualified.

I should be glad if you can tell me the names of the competent authorised examining bodies, whose certificates are accepted by the Local Government Board.—*L. G. W.*

See reply to *D. C.*

---

**Standard Books of Reference.**

"The Nursing Profession: How and Where to Train." 2s. net; post free 2s. 4d.

"Burdett's Official Nursing Directory." 3s. net; post free, 3s. 4d.

"Burdett's Hospitals and Charities." 5s.

"The Nurses' Dictionary of Medical Terms." 2s.

"Burdett's Series of Nursing Text-Books." 1s. each.

"A Handbook for Nurses." (Illustrated). 5s.

"Nursing: Its Theory and Practice." New Edition. 3s. 6d.

"Helps in Sickness and to Health." Fifteenth Thousand. 5s.

"The Physiological Feeding of Infants." 1s.

"The Physiological Nursery Chart." 1s.; post free, 1s. 3d.

"Hospital Expenditure: The Commissariat." 2s. 6d.

All these are published by the Scientific Press, Ltd., and may be obtained through any bookseller or direct from the publishers 28 and 29 Southampton Street, London, W.C.

## Figures and Tables

**Fig. 13 f1:**
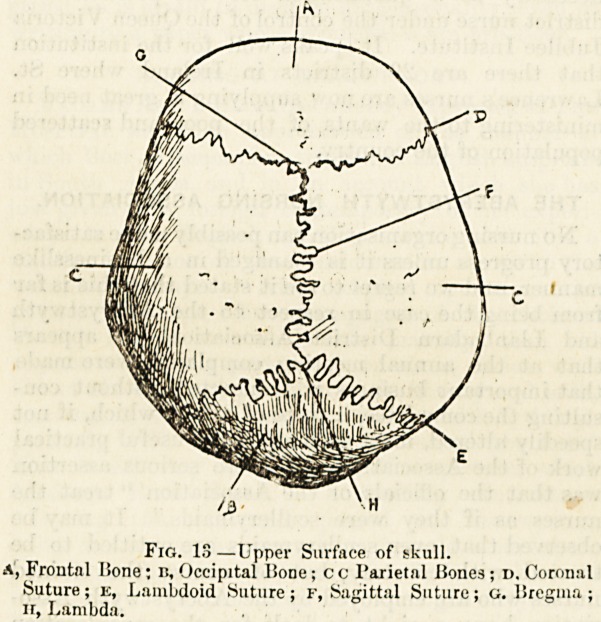


**Fig. 14. f2:**